# Supplementary material for: A Likelihood Approach for Real-Time Calibration of Stochastic Compartmental Epidemic Models
Source: PLoS Comput Biol. 2017 Jan 17;13(1):e1005257. doi: 10.1371/journal.pcbi.1005257 (PMC5240920; doi:10.1371/journal.pcbi.1005257)
Supplement: S1 File — (TAR.GZ) [file pcbi.1005257.s014.tar.gz › HSPH_Online-SI-Revision/Additions/MSS11/n100-extreme_noisy/n100-extreme_noisy_NEW2_table4.pdf]

| | | | | | | | | | | | | | | | | | | | | | | | | | | |
|---|---|---|---|---|---|---|---|---|---|---|---|---|---|---|---|---|---|---|---|---|---|---|---|---|---|---|
| 1 | newly 3 weeks spec | 2278.3 | 2519.19 | {2460., 2586.} | 10.573 | 0.0435611 | newly 3 weeks cumul | 9885.83 | 10284.2 | {10186., 10375.} | 4.0292 | 0.0171438 | newly 5 weeks spec | 1200.81 | 1383.36 | {1327., 1425.} | 15.2018 | 0.061293 | newly 5 weeks cumul | 12674.4 | 13546.6 | {13434., 13670.} | 6.88162 | 0.0288929 | newly 50 weeks cumul | 18298.5 | 17447.9 | {17309., 17577.} | 4.64878 | 0.0206816 |
| 2 | newly 3 weeks spec | 2435. | 2313.69 | {2250., 2386.} | 4.99507 | 0.0223736 | newly 3 weeks cumul | 9225. | 9183.67 | {9072., 9301.} | 0.870352 | 0.00379509 | newly 5 weeks spec | 1337. | 1341.23 | {1293., 1396.} | 2.31638 | 0.0100245 | newly 5 weeks cumul | 12421. | 12300.3 | {12176., 12451.} | 1.12841 | 0.00493512 | newly 46 weeks cumul | 16331. | 16293.2 | {16118., 16490.} | 0.753046 | 0.00327758 |
| 3 | newly 3 weeks spec | 1444. | 1217.7 | {1174., 1257.} | 15.6717 | 0.0741997 | newly 3 weeks cumul | 6755. | 5913.23 | {5809., 6010.} | 12.4614 | 0.0578391 | newly 5 weeks spec | 609. | 507.35 | {469., 532.} | 16.6913 | 0.0798642 | newly 5 weeks cumul | 8307. | 7209.75 | {7070., 7337.} | 13.2087 | 0.0615642 | newly 51 weeks cumul | 9551. | 8122.6 | {7988., 8251.} | 14.9555 | 0.0703915 |
| 4 | newly 3 weeks spec | 766. | 875.98 | {836., 921.} | 14.3577 | 0.0579256 | newly 3 weeks cumul | 4576. | 4923.09 | {4841., 5006.} | 7.68116 | 0.0321053 | newly 5 weeks spec | 254. | 293.32 | {273., 312.} | 15.5433 | 0.0620082 | newly 5 weeks cumul | 5299. | 5722.54 | {5616., 5820.} | 8.08303 | 0.0337199 | newly 55 weeks cumul | 5634. | 6128.09 | {6027., 6238.} | 8.86635 | 0.0368591 |
| 5 | newly 3 weeks spec | 1978. | 2294.37 | {2229., 2362.} | 15.9944 | 0.0643316 | newly 3 weeks cumul | 9313. | 10155.9 | {10014., 10259.} | 9.051 | 0.0376118 | newly 5 weeks spec | 894. | 1105.37 | {1052., 1153.} | 23.6432 | 0.0919071 | newly 5 weeks cumul | 11542. | 12863.3 | {12702., 12992.} | 11.4475 | 0.0470546 | newly 52 weeks cumul | 13327. | 15300.1 | {15112., 15461.} | 14.8052 | 0.0599458 |
| 6 | newly 3 weeks spec | 2370. | 2485.23 | {2413., 2547.} | 4.91435 | 0.0207537 | newly 3 weeks cumul | 9890. | 10398.4 | {10284., 10493.} | 5.14004 | 0.0217543 | newly 5 weeks spec | 1132. | 1309.24 | {1259., 1347.} | 15.6572 | 0.0630104 | newly 5 weeks cumul | 12727. | 13525.6 | {13402., 13654.} | 6.27477 | 0.0264202 | newly 50 weeks cumul | 15600. | 16961.1 | {16829., 17098.} | 8.72468 | 0.0363202 |
| 7 | newly 3 weeks spec | 1951. | 2502.33 | {2445., 2606.} | 28.2588 | 0.107236 | newly 3 weeks cumul | 8547. | 10243.8 | {10190., 10474.} | 19.8584 | 0.0781931 | newly 5 weeks spec | 968. | 1362.94 | {1327., 1434.} | 40.8946 | 0.147273 | newly 5 weeks cumul | 10776. | 13459. | {13462., 13769.} | 24.8977 | 0.095918 | newly 48 weeks cumul | 12929. | 17264.4 | {17326., 17710.} | 33.5323 | 0.124566 |
| 8 | newly 3 weeks spec | 2287. | 2488.35 | {2430., 2539.} | 8.80411 | 0.0365572 | newly 3 weeks cumul | 9859. | 10401.9 | {10264., 10510.} | 5.50644 | 0.0232617 | newly 5 weeks spec | 1178. | 1314.43 | {1263., 1362.} | 11.5815 | 0.0474232 | newly 5 weeks cumul | 12762. | 13529.7 | {13380., 13659.} | 6.01567 | 0.0253576 | newly 50 weeks cumul | 15717. | 16962.7 | {16837., 17113.} | 7.92549 | 0.0331144 |
| 9 | newly 3 weeks spec | 2292. | 2726.59 | {2656., 2799.} | 18.9612 | 0.0753133 | newly 3 weeks cumul | 10287. | 11666.9 | {11545., 11771.} | 13.414 | 0.0546562 | newly 5 weeks spec | 1160. | 1385.15 | {1339., 1426.} | 19.4095 | 0.0768878 | newly 5 weeks cumul | 13098. | 14996. | {14867., 15121.} | 14.4911 | 0.0587631 | newly 50 weeks cumul | 15554. | 18286.6 | {18101., 18431.} | 17.5687 | 0.0702812 |
| 10 | newly 3 weeks spec | 296. | 349.8 | {308., 388.} | 18.5 | 0.0722639 | newly 3 weeks cumul | 1966. | 2238.98 | {2128., 2352.} | 13.9013 | 0.0561699 | newly 5 weeks spec | 92. | 95.04 | {74., 123.} | 15.5435 | 0.0658074 | newly 5 weeks cumul | 2237. | 2513.05 | {2368., 2663.} | 12.3947 | 0.0503053 | newly 54 weeks cumul | 2337. | 2613.88 | {2455., 2779.} | 11.9375 | 0.0485285 |
| 11 | newly 3 weeks spec | 2110. | 2218.87 | {2152., 2297.} | 5.15972 | 0.0217112 | newly 3 weeks cumul | 9055. | 9103.85 | {8967., 9218.} | 0.995583 | 0.00430093 | newly 5 weeks spec | 1137. | 1203.85 | {1144., 1245.} | 6.0431 | 0.0253194 | newly 5 weeks cumul | 11827. | 11954.4 | {11805., 12096.} | 1.19405 | 0.00514158 | newly 48 weeks cumul | 14734. | 15143.1 | {14910., 15347.} | 2.77623 | 0.0118681 |
| 12 | newly 3 weeks spec | 2018. | 2331.76 | {2269., 2391.} | 15.5481 | 0.0626706 | newly 3 weeks cumul | 9115. | 9971.72 | {9880., 10065.} | 9.39901 | 0.039001 | newly 5 weeks spec | 959. | 1200.54 | {1155., 1255.} | 25.1867 | 0.0973826 | newly 5 weeks cumul | 11517. | 12851.5 | {12756., 12952.} | 11.5871 | 0.0476059 | newly 49 weeks cumul | 13641. | 15756.7 | {15645., 15909.} | 15.5099 | 0.0626103 |
| 13 | newly 3 weeks spec | 3118. | 2699.71 | {2425., 3378.} | 17.1446 | 0.0818418 | newly 3 weeks cumul | 12156. | 10865.5 | {10069., 13134.} | 14.2296 | 0.0664071 | newly 5 weeks spec | 2008. | 1515.16 | {1314., 2039.} | 25.3147 | 0.132474 | newly 5 weeks cumul | 16586. | 14431.2 | {13248., 17807.} | 16.3607 | 0.0777879 | newly 47 weeks cumul | 22880. | 18718.9 | {16802., 24421.} | 21.1549 | 0.104911 |
| 14 | newly 3 weeks spec | 249. | 1268.06 | {1203., 1327.} | 409.261 | 0.706629 | newly 3 weeks cumul | 1934. | 6544.96 | {6436., 6663.} | 238.416 | 0.529405 | newly 5 weeks spec | 37. | 479.84 | {441., 514.} | 1196.86 | 1.11213 | newly 5 weeks cumul | 2082. | 7802.43 | {7660., 7935.} | 274.756 | 0.573704 | newly 52 weeks cumul | 2131. | 8548.61 | {8395., 8678.} | 301.155 | 0.603268 |
| 15 | newly 3 weeks spec | 658. | 739.26 | {684., 784.} | 12.3495 | 0.0500894 | newly 3 weeks cumul | 3705. | 4092.61 | {3963., 4199.} | 10.4618 | 0.0431105 | newly 5 weeks spec | 197. | 254.96 | {231., 286.} | 29.4213 | 0.110662 | newly 5 weeks cumul | 4246. | 4783.97 | {4647., 4911.} | 12.67 | 0.0517018 | newly 52 weeks cumul | 4525. | 5142.45 | {4976., 5286.} | 13.6453 | 0.0554241 |
| 16 | newly 3 weeks spec | 902. | 1523.76 | {1477., 1581.} | 68.9313 | 0.22757 | newly 3 weeks cumul | 5020. | 7424.53 | {7331., 7522.} | 47.899 | 0.169941 | newly 5 weeks spec | 342. | 637.94 | {595., 674.} | 86.5322 | 0.270267 | newly 5 weeks cumul | 5877. | 9051.21 | {8939., 9170.} | 54.0107 | 0.18753 | newly 52 weeks cumul | 6347. | 10209.6 | {10070., 10339.} | 60.8576 | 0.20642 |
| 17 | newly 3 weeks spec | 2633. | 2877.01 | {2812., 2941.} | 9.26738 | 0.0384235 | newly 3 weeks cumul | 10424. | 11183.5 | {11065., 11288.} | 7.28617 | 0.0305322 | newly 5 weeks spec | 1461. | 1719.74 | {1659., 1778.} | 17.7098 | 0.0706701 | newly 5 weeks cumul | 13884. | 15124.6 | {14999., 15237.} | 8.93518 | 0.0371604 | newly 47 weeks cumul | 18108. | 20651.6 | {20511., 20808.} | 14.0469 | 0.057077 |
| 18 | newly 3 weeks spec | 1965. | 2067.59 | {1994., 2136.} | 5.32875 | 0.0224043 | newly 3 weeks cumul | 8760. | 9145.51 | {9023., 9292.} | 4.4008 | 0.018672 | newly 5 weeks spec | 847. | 983.29 | {932., 1027.} | 16.0909 | 0.0644821 | newly 5 weeks cumul | 10903. | 11565.2 | {11394., 11736.} | 6.07328 | 0.0255734 | newly 48 weeks cumul | 12657. | 13642. | {13427., 13858.} | 7.78218 | 0.0325151 |
| 19 | newly 3 weeks spec | 2260. | 2731.45 | {2666., 2793.} | 20.8606 | 0.0822141 | newly 3 weeks cumul | 9867. | 11680.2 | {11576., 11789.} | 18.3759 | 0.0732514 | newly 5 weeks spec | 1026. | 1380.75 | {1337., 1436.} | 34.576 | 0.128785 | newly 5 weeks cumul | 12362. | 14996.1 | {14873., 15120.} | 21.3083 | 0.0838808 | newly 50 weeks cumul | 14680. | 18290.7 | {18132., 18479.} | 24.596 | 0.0954942 |
| 20 | newly 3 weeks spec | 351. | 1110.02 | {1066., 1152.} | 216.245 | 0.499848 | newly 3 weeks cumul | 2657. | 6112.41 | {6033., 6187.} | 130.049 | 0.361794 | newly 5 weeks spec | 80. | 387.65 | {358., 418.} | 384.563 | 0.684667 | newly 5 weeks cumul | 2903. | 7157. | {7050., 7256.} | 146.538 | 0.391857 | newly 55 weeks cumul | 3010. | 7702.29 | {7596., 7807.} | 155.89 | 0.408028 |
| 21 | newly 3 weeks spec | 1266. | 1511.63 | {1447., 1575.} | 19.4021 | 0.0767952 | newly 3 weeks cumul | 6452. | 7307.72 | {7202., 7424.} | 13.2629 | 0.0540592 | newly 5 weeks spec | 504. | 637.74 | {598., 676.} | 26.5357 | 0.101676 | newly 5 weeks cumul | 7741. | 8930.8 | {8800., 9057.} | 15.3701 | 0.062061 | newly 52 weeks cumul | 8578. | 10076.6 | {9921., 10222.} | 17.4697 | 0.0698945 |
| 22 | newly 3 weeks spec | 1097. | 1541.93 | {1485., 1600.} | 40.5588 | 0.147692 | newly 3 weeks cumul | 5662. | 7466.41 | {7379., 7573.} | 31.8688 | 0.120119 | newly 5 weeks spec | 414. | 652.3 | {615., 684.} | 57.5604 | 0.197089 | newly 5 weeks cumul | 6768. | 9114.03 | {9008., 9224.} | 34.6636 | 0.129233 | newly 53 weeks cumul | 7400. | 10295.7 | {10162., 10413.} | 39.1316 | 0.143407 |
| 23 | newly 3 weeks spec | 2274. | 2644.27 | {2574., 2712.} | 16.2828 | 0.0654246 | newly 3 weeks cumul | 9195. | 10364.2 | {10263., 10449.} | 12.7152 | 0.0519711 | newly 5 weeks spec | 1192. | 1553.01 | {1499., 1598.} | 30.2861 | 0.114769 | newly 5 weeks cumul | 12094. | 13949.9 | {13838., 14048.} | 15.3455 | 0.0619932 | newly 46 weeks cumul | 15344. | 18925. | {18788., 19061.} | 23.3379 | 0.0910897 |
| 24 | newly 3 weeks spec | 2818. | 2616.22 | {2547., 2689.} | 7.1604 | 0.0323563 | newly 3 weeks cumul | 11319. | 10532.3 | {10432., 10631.} | 6.95061 | 0.0313004 | newly 5 weeks spec | 1477. | 1463.92 | {1414., 1512.} | 2.31686 | 0.0101462 | newly 5 weeks cumul | 14831. | 13966.3 | {13823., 14081.} | 5.83069 | 0.0261018 | newly 46 weeks cumul | 19249. | 18201.3 | {18028., 18396.} | 5.44314 | 0.0243199 |
| 25 | newly 3 weeks spec | 1110. | 1939.87 | {1890., 1994.} | 74.7631 | 0.242356 | newly 3 weeks cumul | 5663. | 8453.83 | {8344., 8556.} | 49.2818 | 0.173986 | newly 5 weeks spec | 432. | 981.44 | {935., 1022.} | 127.185 | 0.356146 | newly 5 weeks cumul | 6746. | 10820.1 | {10692., 10951.} | 60.393 | 0.20517 | newly 49 weeks cumul | 7332. | 13129.8 | {13000., 13276.} | 79.0749 | 0.253021 |
| 26 | newly 3 weeks spec | 2919. | 2871.99 | {2805., 2938.} | 1.9421 | 0.00855291 | newly 3 weeks cumul | 11387. | 11182.9 | {11069., 11290.} | 1.79213 | 0.00786674 | newly 5 weeks spec | 1758. | 1719.37 | {1668., 1780.} | 2.82423 | 0.012479 | newly 5 weeks cumul | 15461. | 15120.8 | {14985., 15249.} | 2.20018 | 0.00967168 | newly 47 weeks cumul | 21236. | 20666.1 | {20469., 20843.} | 2.68389 | 0.0118236 |
| 27 | newly 3 weeks spec | 2411. | 2156.96 | {2076., 2228.} | 10.5367 | 0.0485192 | newly 3 weeks cumul | 9503. | 8780.7 | {8506., 8933.} | 7.60076 | 0.0343972 | newly 5 weeks spec | 1418. | 1199.43 | {1142., 1253.} | 15.414 | 0.0729719 | newly 5 weeks cumul | 12786. | 11602.2 | {11307., 11792.} | 9.25856 | 0.0422493 | newly 48 weeks cumul | 17179. | 14968.5 | {14641., 15179.} | 12.8677 | 0.0598623 |
| 28 | newly 3 weeks spec | 2066. | 1547.76 | {1493., 1601.} | 25.0842 | 0.125571 | newly 3 weeks cumul | 9289. | 7251.35 | {7135., 7354.} | 21.9362 | 0.107585 | newly 5 weeks spec | 1123. | 677.5 | {634., 721.} | 39.6705 | 0.22001 | newly 5 weeks cumul | 12043. | 8952.64 | {8816., 9094.} | 25.661 | 0.128817 | newly 52 weeks cumul | 14773. | 10261.7 | {10102., 10438.} | 30.5377 | 0.15829 |
| 29 | newly 3 weeks spec | 2511. | 2564.81 | {2454., 2672.} | 2.9554 | 0.0125987 | newly 3 weeks cumul | 10166. | 10364.4 | {10170., 10549.} | 2.01977 | 0.00864867 | newly 5 weeks spec | 1397. | 1415.09 | {1338., 1505.} | 3.48533 | 0.0149295 | newly 5 weeks cumul | 13376. | 13696. | {13434., 14029.} | 2.4147 | 0.0103099 | newly 46 weeks cumul | 17318. | 17593.2 | {17098., 18270.} | 2.21175 | 0.00943392 |
| 30 | newly 3 weeks spec | 1594. | 1598.89 | {1543., 1652.} | 2.19887 | 0.00953743 | newly 3 weeks cumul | 7832. | 7897.1 | {7790., 7983.} | 1.05899 | 0.00456919 | newly 5 weeks spec | 651. | 651.65 | {616., 689.} | 3.5745 | 0.015531 | newly 5 weeks cumul | 9505. | 9572.11 | {9453., 9680.} | 0.934771 | 0.004036 | newly 55 weeks cumul | 10576. | 10721.4 | {10605., 10847.} | 1.45745 | 0.00627094 |
| 31 | newly 3 weeks spec | 771. | 374.89 | {345., 404.} | 51.3761 | 0.314218 | newly 3 weeks cumul | 4574. | 2752.37 | {2668., 2839.} | 39.8258 | 0.220726 | newly 5 weeks spec | 189. | 85.73 | {68., 103.} | 54.6402 | 0.348245 | newly 5 weeks cumul | 5139. | 3016.39 | {2909., 3127.} | 41.304 | 0.231555 | newly 56 weeks cumul | 5392. | 3097.67 | {2990., 3210.} | 42.5506 | 0.240897 |
| 32 | newly 3 weeks spec | 858. | 495.3 | {447., 538.} | 42.2727 | 0.23959 | newly 3 weeks cumul | 4649. | 3123.71 | {2987., 3238.} | 32.809 | 0.172903 | newly 5 weeks spec | 288. | 140.9 | {113., 169.} | 51.0764 | 0.315548 | newly 5 weeks cumul | 5438. | 3532.2 | {3370., 3667.} | 35.046 | 0.18768 | newly 53 weeks cumul | 5832. | 3689.59 | {3512., 3858.} | 36.7354 | 0.199177 |
| 33 | newly 3 weeks spec | 3068. | 2754.46 | {2681., 2824.} | 10.2197 | 0.0469125 | newly 3 weeks cumul | 12287. | 11429.7 | {11336., 11546.} | 6.9777 | 0.0314251 | newly 5 weeks spec | 1670. | 1456.43 | {1398., 1498.} | 12.7886 | 0.0596077 | newly 5 weeks cumul | 16122. | 14890.6 | {14752., 15025.} | 7.63832 | 0.0345192 | newly 51 weeks cumul | 20713. | 18599.1 | {18439., 18779.} | 10.2057 | 0.0467619 |
| 34 | newly 3 weeks spec | 2614. | 2851.55 | {2772., 2929.} | 9.08761 | 0.0376819 | newly 3 weeks cumul | 10179. | 10933.4 | {10795., 11070.} | 7.41173 | 0.0310321 | newly 5 weeks spec | 1528. | 1720.09 | {1665., 1771.} | 12.5713 | 0.051303 | newly 5 weeks cumul | 13712. | 14878.4 | {14710., 15056.} | 8.50656 | 0.0354391 | newly 46 weeks cumul | 18212. | 20328. | {20084., 20579.} | 11.6187 | 0.0477183 |
| 35 | newly 3 weeks spec | 2810. | 2352.47 | {2277., 2417.} | 16.2822 | 0.0772937 | newly 3 weeks cumul | 11877. | 10354.1 | {10225., 10464.} | 12.822 | 0.0596116 | newly 5 weeks spec | 1428. | 1131.13 | {1080., 1171.} | 20.7892 | 0.101426 | newly 5 weeks cumul | 15254. | 13121.9 | {12996., 13249.} | 13.9774 | 0.0653992 | newly 51 weeks cumul | 18586. | 15615.3 | {15463., 15801.} | 15.9833 | 0.07565 |
| 36 | newly 3 weeks spec | 2377. | 2573.51 | {2456., 2696.} | 8.26714 | 0.0342497 | newly 3 weeks cumul | 9678. | 10368.2 | {10073., 10688.} | 7.13154 | 0.0297942 | newly 5 weeks spec | 1295. | 1439.18 | {1349., 1554.} | 11.1336 | 0.0452309 | newly 5 weeks cumul | 12751. | 13744.8 | {13331., 14264.} | 7.79406 | 0.0324277 | newly 49 weeks cumul | 16300. | 17794.7 | {17067., 18705.} | 9.17018 | 0.0377741 |
| 37 | newly 3 weeks spec | 918. | 884.59 | {799., 915.} | 10.3736 | 0.0437566 | newly 3 weeks cumul | 5030. | 4948.78 | {4710., 4950.} | 6.45408 | 0.0267985 | newly 5 weeks spec | 306. | 301.38 | {254., 318.} | 12.6405 | 0.0511614 | newly 5 weeks cumul | 5864. | 5767.95 | {5446., 5735.} | 7.04656 | 0.0290686 | newly 54 weeks cumul | 6382. | 6192.95 | {5820., 6145.} | 8.7482 | 0.0365781 |
| 38 | newly 3 weeks spec | 286. | 1029.77 | {989., 1075.} | 260.059 | 0.556143 | newly 3 weeks cumul | 2079. | 5598.64 | {5507., 5714.} | 169.295 | 0.430188 | newly 5 weeks spec | 76. | 366.37 | {348., 389.} | 382.066 | 0.682589 | newly 5 weeks cumul | 2297. | 6580.95 | {6477., 6684.} | 186.502 | 0.45709 | newly 54 weeks cumul | 2347. | 7113.17 | {6985., 7241.} | 203.075 | 0.481511 |
| 39 | newly 3 weeks spec | 1569. | 1875.05 | {1808., 1940.} | 19.5061 | 0.0772041 | newly 3 weeks cumul | 6961. | 8083.25 | {7931., 8219.} | 16.122 | 0.0648785 | newly 5 weeks spec | 719. | 925.19 | {876., 978.} | 28.6773 | 0.10903 | newly 5 weeks cumul | 8738. | 10330.9 | {10157., 10494.} | 18.2291 | 0.0726854 | newly 48 weeks cumul | 10021. | 12423.3 | {12167., 12645.} | 23.9729 | 0.0932758 |
| 40 | newly 3 weeks spec | 2069. | 1676.43 | {1629., 1734.} | 18.9739 | 0.0915112 | newly 3 weeks cumul | 8421. | 7284.1 | {7144., 7401.} | 13.5008 | 0.0630265 | newly 5 weeks spec | 1045. | 830.17 | {780., 888.} | 20.5579 | 0.100391 | newly 5 weeks cumul | 10908. | 9301.93 | {9147., 9465.} | 14.7238 | 0.0692093 | newly 49 weeks cumul | 13560. | 11245. | {11006., 11477.} | 17.0721 | 0.0813506 |
| 41 | newly 3 weeks spec | 1243. | 1736.47 | {1668., 1807.} | 39.6999 | 0.144963 | newly 3 weeks cumul | 5945. | 7543.57 | {7407., 7678.} | 26.8893 | 0.103382 | newly 5 weeks spec | 608. | 860.19 | {805., 905.} | 41.4786 | 0.150227 | newly 5 weeks cumul | 7464. | 9620.98 | {9472., 9788.} | 28.8984 | 0.110205 | newly 50 weeks cumul | 8541. | 11562.7 | {11295., 11786.} | 35.3791 | 0.131498 |
| 42 | newly 3 weeks spec | 457. | 342.52 | {305., 382.} | 25.0503 | 0.127083 | newly 3 weeks cumul | 2671. | 2207.22 | {2097., 2306.} | 17.3635 | 0.0831615 | newly 5 weeks spec | 82. | 93.46 | {76., 115.} | 18.5366 | 0.0727777 | newly 5 weeks cumul | 2987. | 2478.17 | {2347., 2593.} | 17.0348 | 0.0814729 | newly 54 weeks cumul | 3092. | 2582.27 | {2439., 2697.} | 16.4854 | 0.0786527 |
| 43 | newly 3 weeks spec | 2726. | 3247.28 | {3165., 3319.} | 19.1225 | 0.0759249 | newly 3 weeks cumul | 11008. | 12731.6 | {12608., 12837.} | 15.6573 | 0.0631624 | newly 5 weeks spec | 1377. | 1919.51 | {1864., 1974.} | 39.398 | 0.144139 | newly 5 weeks cumul | 14359. | 17151. | {16992., 17261.} | 19.444 | 0.0771569 | newly 47 weeks cumul | 18000. | 23142.6 | {22984., 23284.} | 28.5702 | 0.109135 |
| 44 | newly 3 weeks spec | 1026. | 1422.67 | {1382., 1472.} | 38.6618 | 0.141828 | newly 3 weeks cumul | 5335. | 6737.58 | {6645., 6826.} | 26.2902 | 0.101349 | newly 5 weeks spec | 393. | 623.28 | {593., 658.} | 58.5954 | 0.199918 | newly 5 weeks cumul | 6350. | 8293.45 | {8214., 8390.} | 30.6055 | 0.115945 | newly 53 weeks cumul | 7041. | 9496.28 | {9392., 9623.} | 34.8712 | 0.129901 |
| 45 | newly 3 weeks spec | 374. | 741.89 | {689., 788.} | 98.3663 | 0.296958 | newly 3 weeks cumul | 2546. | 4094.62 | {4000., 4199.} | 60.8256 | 0.206278 | newly 5 weeks spec | 135. | 252.64 | {221., 283.} | 87.1407 | 0.270303 | newly 5 weeks cumul | 2913. | 4785.54 | {4650., 4913.} | 64.2822 | 0.215495 | newly 52 weeks cumul | 3058. | 5146.53 | {4995., 5302.} | 68.2973 | 0.225962 |
| 46 | newly 3 weeks spec | 1322. | 1857.66 | {1804., 1925.} | 40.5189 | 0.147608 | newly 3 weeks cumul | 7028. | 8962.06 | {8852., 9090.} | 27.5194 | 0.105551 | newly 5 weeks spec | 520. | 786.73 | {741., 833.} | 51.2942 | 0.179441 | newly 5 weeks cumul | 8416. | 10955.2 | {10822., 11098.} | 30.171 | 0.114492 | newly 52 weeks cumul | 9218. | 12368.9 | {12228., 12518.} | 34.1815 | 0.127671 |
| 47 | newly 3 weeks spec | 877. | 852. | {807., 894.} | 3.84036 | 0.0171323 | newly 3 weeks cumul | 5116. | 4831.03 | {4726., 4937.} | 5.57017 | 0.0249533 | newly 5 weeks spec | 280. | 284.49 | {256., 312.} | 6.34643 | 0.0271652 | newly 5 weeks cumul | 5861. | 5608.94 | {5485., 5730.} | 4.30063 | 0.0191566 | newly 54 weeks cumul | 6385. | 5996.11 | {5856., 6138.} | 6.09068 | 0.0273622 |
| 48 | newly 3 weeks spec | 1336. | 1173.84 | {1128., 1217.} | 12.1377 | 0.0565273 | newly 3 weeks cumul | 7014. | 6259.7 | {6167., 6376.} | 10.7542 | 0.0495306 | newly 5 weeks spec | 498. | 422.78 | {395., 455.} | 15.1044 | 0.0721503 | newly 5 weeks cumul | 8264. | 7394.23 | {7283., 7503.} | 10.5248 | 0.0484377 | newly 54 weeks cumul | 8948. | 8035.17 | {7929., 8170.} | 10.2015 | 0.0468965 |
| 49 | newly 3 weeks spec | 215. | 369.23 | {340., 391.} | 71.7349 | 0.23419 | newly 3 weeks cumul | 1583. | 2479.77 | {2408., 2542.} | 56.65 | 0.194831 | newly 5 weeks spec | 43. | 98.59 | {84., 112.} | 129.279 | 0.357755 | newly 5 weeks cumul | 1735. | 2769.59 | {2697., 2847.} | 59.6305 | 0.203017 | newly 56 weeks cumul | 1768. | 2873.41 | {2802., 2938.} | 62.5232 | 0.21081 |
| 50 | newly 3 weeks spec | 2185. | 2248.06 | {2164., 2318.} | 3.30892 | 0.0140621 | newly 3 weeks cumul | 9176. | 9152.36 | {8916., 9303.} | 1.18047 | 0.0051534 | newly 5 weeks spec | 1236. | 1251.16 | {1194., 1309.} | 3.14239 | 0.0134757 | newly 5 weeks cumul | 12158. | 12082.8 | {11744., 12285.} | 1.16376 | 0.00510458 | newly 47 weeks cumul | 15907. | 15584.1 | {15107., 15835.} | 2.04086 | 0.00901051 |
